# Split-QF System for Fine-Tuned Transgene Expression in *Drosophila*

**DOI:** 10.1534/genetics.119.302034

**Published:** 2019-03-12

**Authors:** Olena Riabinina, Samuel W. Vernon, Barry J. Dickson, Richard A. Baines

**Affiliations:** * Division of Neuroscience and Experimental Psychology, School of Biological Sciences, Faculty of Biology, Medicine and Health, Manchester Academic Health Science Centre, University of Manchester, M13 9PL, United Kingdom; † Janelia Research Campus, Howard Hughes Medical Institute, Ashburn, Virginia 21407

**Keywords:** Q-system, quinic acid, split-GAL4, split-LexA

## Abstract

The Q-system is a binary expression system that works well across species. Here, we report the development and demonstrate the applications of a split-QF system that drives strong expression in *Drosophila*, is repressible by QS, and is inducible by a small nontoxic molecule (quinic acid). The split-QF system is fully compatible with existing split-GAL4 and split-LexA lines, thus greatly expanding the range of possible advanced intersectional experiments and anatomical, physiological, and behavioral assays in *Drosophila*, and in other organisms.

BINARY expression systems such as GAL4/UAS (Brand and Perrimon 1993), LexA/LexAop (Lai and Lee 2006), and the Q-system (Potter *et al.* 2010; Riabinina *et al.* 2015; Riabinina and Potter 2016) allow the labeling and functional manipulation of genetically defined subsets of cells in *Drosophila*. Several methods have been developed to limit the expression of effectors to small specific subsets of cells (Golic and Lindquist 1989; Lee and Luo 1999; Luan *et al.* 2006; Tirian and Dickson 2017). One of these methods, the split-GAL4 system (Luan *et al.* 2006; Tirian and Dickson 2017; Dionne *et al.* 2018), directs expression of a GAL4 DNA-binding domain (DBD) independently of a GAL4 activation domain (AD). A fully functional GAL4 is reconstituted only where the expression patterns of both subsets overlap. In practice, GAL4AD is often too weak and is replaced by p65AD, or VP16AD, to boost the strength of expression (Tirian and Dickson 2017; Dionne *et al.* 2018).

We reasoned that, since the QF2/QF2^w^ [a weaker version of QF2, with a mutated C-terminal (Riabinina *et al.* 2015)] transactivators of the Q-system are generally stronger than GAL4 (Riabinina *et al.* 2015), the split-QF system may function well in *Drosophila* by coupling QFDBD and QFAD. This approach has been successfully tested in *Caenorhabditis elegans* (Wei *et al.* 2012), but not characterized or used in *Drosophila*. The use of split-QF with QFAD would allow the system to remain both repressible by QS and inducible by quinic acid (QA), in the same manner as the original Q-system. We have also previously developed chimeric GAL4QF and LexAQF transactivators (Riabinina *et al.* 2015), which indicated that QFAD and QF2^w^AD are likely to function with GAL4DBD and LexADBD domains when brought together by leucine zippers.

## Materials and Methods

### Molecular biology

Plasmids were constructed by standard procedures including enzyme digestions, PCR, and subcloning, using the In-Fusion HD Cloning System CE (Takara Bio Europe # 639636). Plasmid inserts were verified by DNA sequencing.

### *nsyb-nls*::*QFAD*::*Zip+* construct:

1. The pattB-QF2-hsp70 plasmid (#46115; Addgene) was digested with *Zra*I and *Eco*RI to remove the Kozak-QF2 sequence.
2. The Kozak-nls sequence was PCR-amplified from pBPp65ADZpUw (#26234; Addgene) with primers 5′-ATC GAC AGC CGA ATT CAA CAT GGA TAA AGC GGA ATT A-3′ (forward) and 5′-ACG GTA TCG ATA GAC GTC CAA TTC GAC CTT TCT CTT C-3′ (reverse).
3. The PCR product was cloned into the digested vector by InFusion cloning.
4. The cloning product was digested with *Zra*I.
5. The QFAD sequence was PCR-amplified from the pattB-QF2-hsp70 plasmid (#46115; Addgene) with primers 5′-AAG GTC GAA TTG GAC GTC CGT CAG TTG GAG CTA A-3′ (forward) and 5′-ACG GTA TCG ATA GAC AGA TCT CTG TTC GTA TGT ATT AAT GTC GGA GAA G-3′ (reverse).
6. The PCR product from step 5 was subcloned into the cloning product from step 4 by InFusion cloning.
7. The product from step 6 was digested with *Bgl*II.
8. The GGGGG-Zip+ sequence was PCR-amplified from pBPp65ADZpUw (#26234; Addgene) with primers 5′-ATA CGA ACA GAG ATC TGG AGG AGG TGG TGG AGG-3′ (forward) and 5′-ATC GAT AGA CAG ATC GGC CGG CCT TAC TTG CCG CCG CC-3′ (reverse).
9. The PCR product from step 8 was subcloned into the digested vector from step 7 by InFusion cloning.
10. The product from step 9 was digested with *Fse*I and *Not*I to remove the hsp70 terminator, and to replace it with the simian virus 40 (SV40) terminator.
11. The SV40 terminator was PCR-amplified from the UAS-LUC-UAS-eYFP plasmid (Lynd and Lycett 2012) with primers 5′-GGC AAG TAA GGC CGG CCG ATC TTT GTG AAG GAA CCT TAC-3′ (forward) and 5′-CCT CGA GCC GCG GCC GCG ATC CAG ACA TGA TAA GAT AC-3′ (reverse).
12. The PCR product from step 11 was subcloned into the vector from step 10 by InFusion cloning.

### *nsyb-nls*::*QF2^w^AD*::*Zip+* construct:

1. The *nsyb-nls*::*QFAD*::*Zip+* construct was digested with *Bgl*II and *Zra*I to remove QFAD.
2. The QF2^w^AD sequence was PCR-amplified from pattb-QF2-hsp70 (#46115; Addgene) with primers 5′-AAG GTC GAA TTG GAC GTC CGT CAG TTG GAG CTC C-3′ (forward) and 5′-CAC CTC CTC CAG ATC TTT CTT CTT TTT GGT ATG TAT TAA TGT CGG AGA AGT TAC ATC C-3′ (reverse).
3. The PCR product from step 2 was cloned into the product from step 1 by InFusion cloning.

### *nsyb-nls*::*p65AD*::*Zip+* construct:

1. The *nsyb-nls*::*QFAD*::*Zip+* construct was digested with *Fse*I and *Zra*I to remove the QFAD::Zip+ sequence.
2. The p65AD::Zip+ sequence was PCR-amplified from pBPp65ADZpUw (#26234; Addgene) with primers 5′-AAG GTC GAA TTG GAC GTC GGA TCC ACG CCG ATG-3′ (forward) and 5′-CTT CAC AAA GAT CGG CCG GCC TTA CTT GCC GCC GCC-3′ (reverse).
3. The PCR product from step 2 was subcloned into the product of step 1 by InFusion cloning.

### *nsyb-nls*::*GAL4AD*::*Zip+* construct:

1. The *nsyb-nls*::*QFAD*::*Zip+* construct was digested with *Bgl*II and *Zra*I to remove QFAD.
2. The GAL4AD sequence was PCR-amplified from pBPGAL4.2Uw-2 (#26227; Addgene) with primers 5′-AAG GTC GAA TTG GAC GTC GCC AAC TTC AAC CAG AGT GG-3′ (forward) and 5′-CAC CTC CTC CAG ATC TCT CCT TCT TTG GGT TCG GTG-3′ (reverse).
3. The PCR product from step 2 was subcloned into the product from step 1 by InFusion cloning.

### *nsyb-Zip−*::*QFDBD* construct:

1. The pattB-QF2-hsp70 plasmid (#46115; Addgene) was digested with *Zra*I and *Eco*RI to remove the Kozak-QF2 sequence.
2. The Kozak-Zip^−^-GGGGGG sequence was PCR-amplified from pBPZpGAL4DBDUw (#26233; Addgene) with primers 5′-ATC GAC AGC CGA ATT CAA CAT GCT GGA GAT CCG C-3′ (forward) and 5′-ACG GTA TCG ATA GAC GTC ACC TCC ACC TCC ACC TCC-3′ (reverse).
3. The PCR product from step 2 was InFusion-subcloned into the product from step 1.
4. The product from step 3 was digested with *Zra*I.
5. QFDBD was PCR-amplified from the pattB-QF2-hsp70 plasmid (#46115; Addgene) with primers 5′-GGA GGT GGA GGT GAC GTC ATG CCA CCC AAG CG-3′ (forward) and 5′-ACG GTA TCG ATA GAC GGC CGG CCT TAG AGG AGG CGG GTA ATG C-3′ (reverse).
6. The PCR product from step 5 was InFusion-subcloned into the product from step 4.
7. The product from step 6 was digested with *Fse*I and *Not*I to remove the hsp70 terminator and replace it with the SV40 terminator.
8. The SV40 terminator was PCR-amplified from the UAS-LUC-UAS-eYFP plasmid (Lynd and Lycett 2012) with primers 5′-CTC CTC TAA GGC CGG CCG ATC TTT GTG AAG GAA CCT TAC-3′ (forward) and 5′-CCT CGA GCC GCG GCC GCG ATC CAG ACA TGA TAA GAT AC-3′ (reverse).
9. The PCR product from step 8 was InFusion-subcloned into the product from step 7.

### New and existing transgenic flies

New transgenic lines were generated by inserting the *nsyb-QFDBD* construct in attp40 (II) and all *nsyb-AD* constructs into attp2 (III). New stocks were deposited to the Bloomington *Drosophila* Stock Centre as ##81281 - 81302.

**Figure 1:**
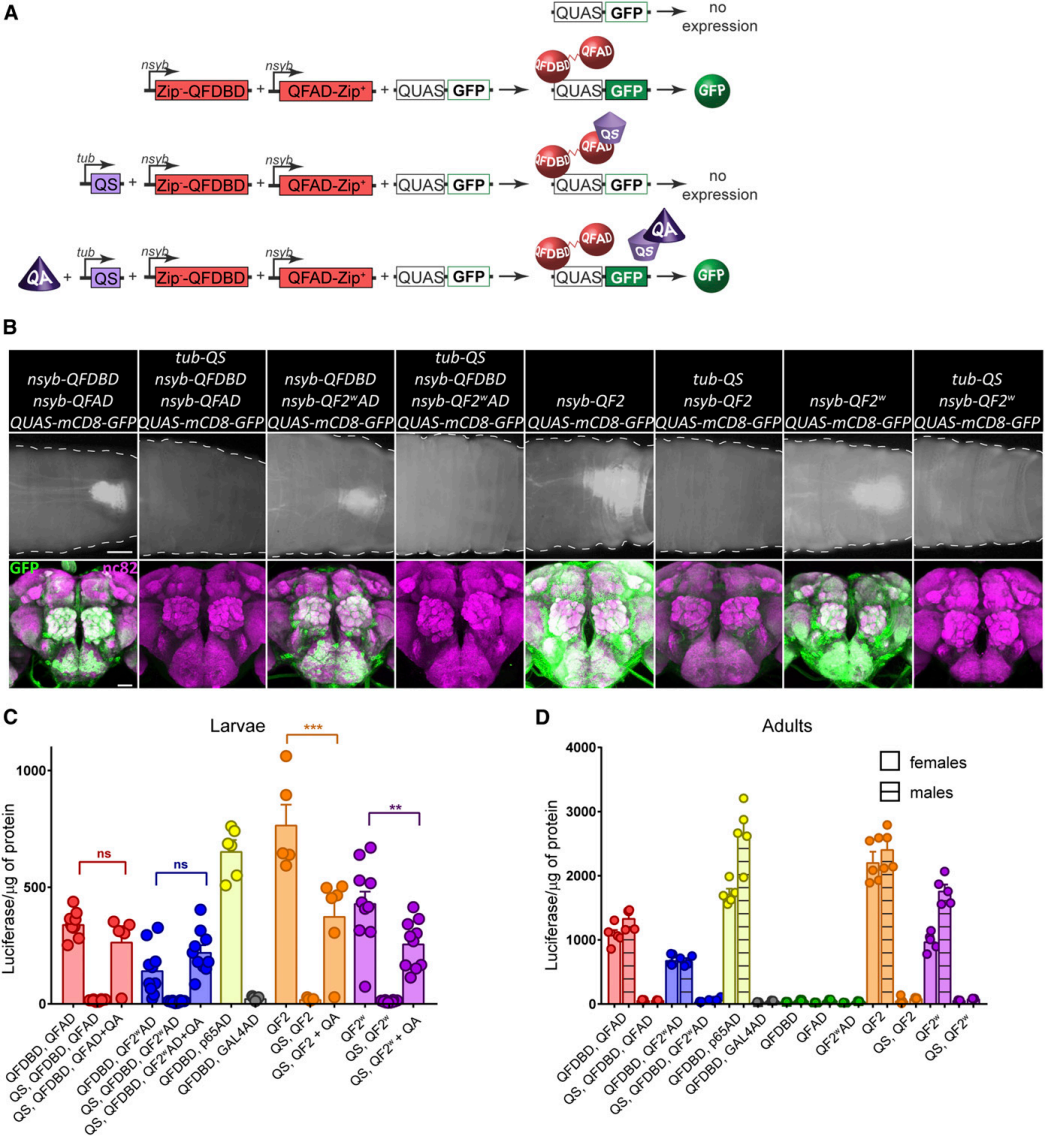
Quantification and validation of split-QF reagents. (A) Schematics of the split-QF system. (B) Pan-neuronal expression of GFP in larval (top; bar, 200 µm) and adult (bottom; bar, 50 µm) CNS by split-QF (first four columns) and the Q-system (last four columns). (C and D) Quantification of split-QF transactivators in larval (C) and adult (D) CNS by a luciferase assay. All split and full-length transactivators were driven by *nsyb*, while QS was driven by *tubulin* (*tub*). Green data points show quantification for *nsyb-QFDBD*, *QUAS-luc*; *nsyb-QFAD*, *QUAS-luc* and *nsyb-QF2^w^AD*, *QUAS-luc* controls.

**Figure 2:**
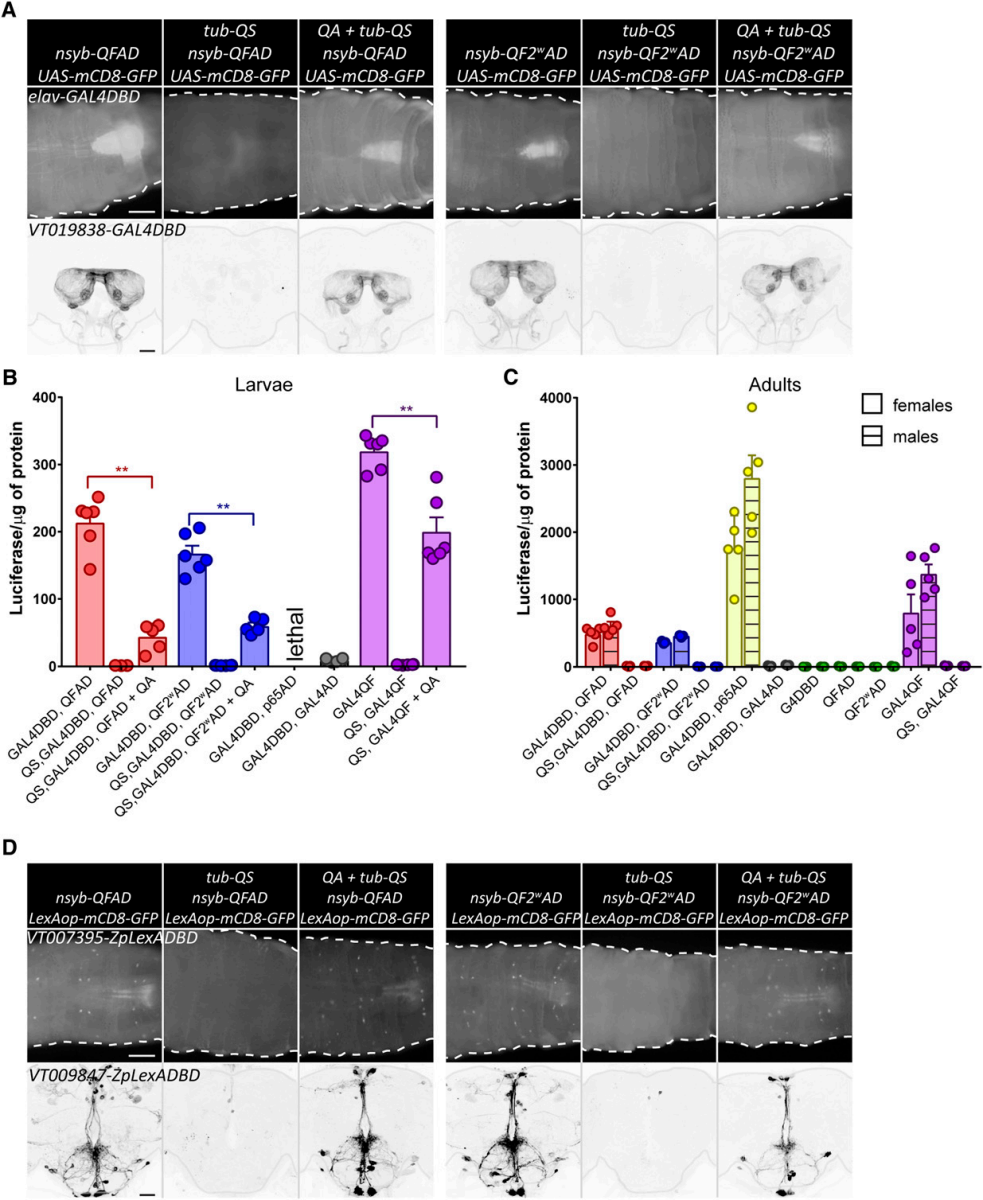
Split-QF, split-GAL4, and split-LexA. (A) Top: expression of GFP in larval CNS, driven by *elav-GAL4DBD* and *nsyb-QFAD* (three left columns), or *nsyb-QF2^w^AD* (three right columns). Second and fifth columns show *tub-QS*-induced repression. Third and sixth columns show recovery of expression in larvae, grown on food with quinic acid (QA). Bar, 200 µm. Bottom: same as top, but driven by *VT019838-GAL4DBD* in adult CNS. Adults were fed with QA for 5 days. Bar, 50 µm. (B) Quantification of relative strength of chimeric split transactivator in larval CNS. Genotypes were *elav-GAL4DBD*, *nsyb-QFAD*, UAS-luc (red) or *elav-GAL4DBD*, *nsyb-QF2^w^AD*, UAS-luc (blue), without (left) or with (middle) *tub-QS* and QA treatment (right). *elav-GAL4DBD*, *nsyb-GAL4AD*, UAS-luc larvae (gray) had very low luciferase levels, while *elav-GAL4DBD*, *nsyb-p65AD*, UAS-luc larvae did not survive. Purple bars show data from *nsyb-GAL4QF*, UAS-luc larvae for comparison. (C) Same as (B), but in adult CNS. Males and females are quantified separately due to significantly different expression levels. Green data points show quantification for *elav-GAL4DBD*, *UAS-luc*; *nsyb-QFAD*, *UAS-luc* and *nsyb-QF2^w^AD*, *UAS-luc* controls. (D) top. Expression of GFP in larval CNS, driven by *VT007395-LexADBD* and *nsyb-QFAD* (three left columns), or *nsyb-QF2^w^AD* (three right columns). Second and fifth columns show tub-QS induced repression. Third and sixth columns show recovery of expression in the larvae, grown on food with QA. Bar, 200 µm. (D) bottom. Same as top, but driven by *VT009847-LexADBD* in adult CNS. Adults were fed with QA for 5 days. Bar, 50 µm.

**Figure 3:**
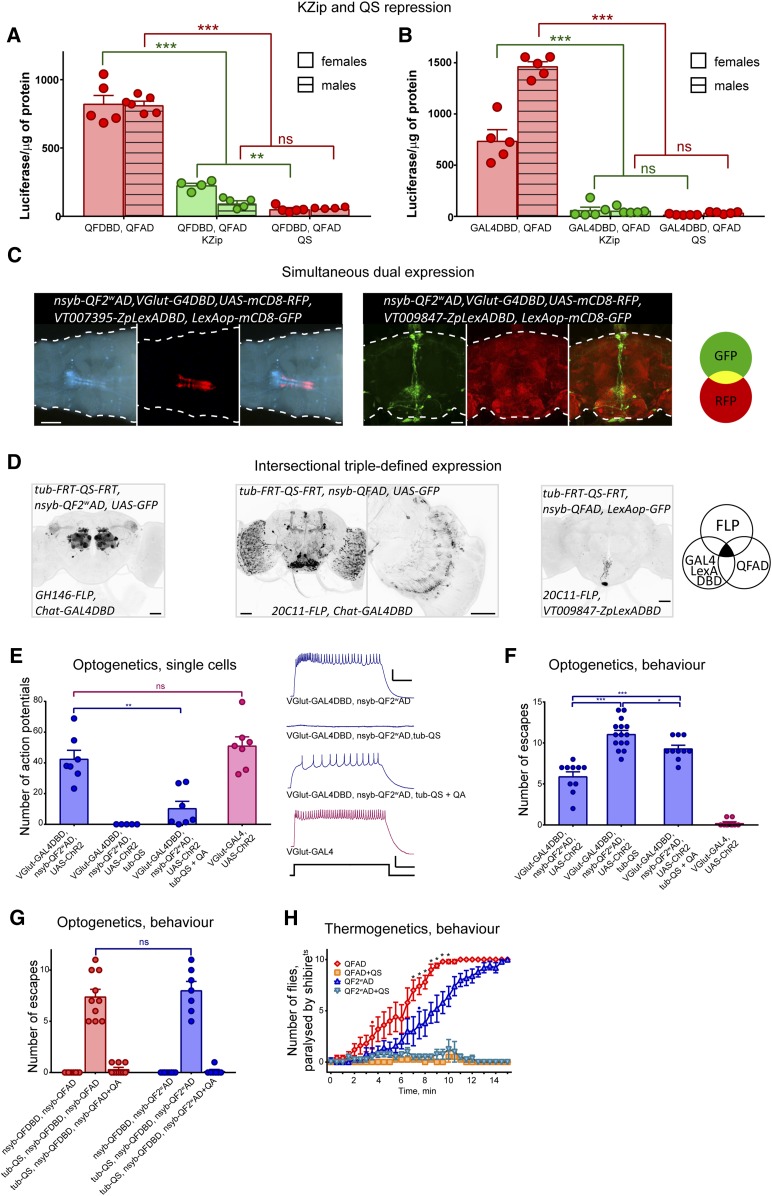
Applications of split-QF. (A and B) Repression of expression by *Killer-Zipper* (Dolan *et al.* 2017) or *tub-QS*. Expression levels were quantified in adult flies using a luciferase assay. Genotypes of flies without repression were *nsyb-QFDBD*, *nsyb-QFAD*, *QUAS-Luc* (A, left) or *elav-GAL4DBD*, *nsyb-QFAD*, *UAS-Luc* (B, left). Killer-Zipper flies were *nsyb-QFDBD*, *nsyb-QFAD*, *nsyb-LexAQF*, *lexAop-KZip+*, *QUAS-Luc* (A, middle, green) or *elav-GAL4DBD*, *nsyb-QFAD*, *nsyb-LexAQF*, *lexAop-KZip+*, *UAS-Luc* (B, middle, green). QS flies were *tub-QS*, *nsyb-QFDBD*, *nsyb-QFAD*, *QUAS-Luc* (A, right) or *tub-QS*, *elav-GAL4DBD*, *nsyb-QFAD*, *UAS-Luc* (B, right). (C) Simultaneous expression of red fluorescent protein (RFP) and GFP in independent neuronal subpopulations in larvae (left; bar, 200 µm) and adults (right; bar, 50 µm), by *QF2^w^AD* forming functional transactivators with *GAL4DBD* and *LexADBD*. (D) Intersectional expression, enabled by QS-repressible *GAL4DBD+QF/QF2^w^AD* and *LexADBD+QFAD* transactivators. GFP is expressed only in cells that: (1) are expressing FLP or are progeny of cells that were expressing FLP; (2) are expressing GAL4DBD or LexADBD; and (3) are expressing QFAD or QF2^w^AD. Third panel shows a zoomed-in image of the z-stack of the brain, shown in the second panel. Bar, 50 µm. (E) Whole-cell patch-clamp recordings from aCC/PR2 motoneurons in third-instar larvae of indicated genotypes, raised on food supplemented with all-trans retinal. Depolarization was elicited by blue light. Example traces are shown on the right. Bars (traces: 10 mV/100 msec, stimulus: 2 V/100 msec). (F) Escape assay of larvae with the same genotypes as in (E). Each larva was given 2 min to escape from a 113 mm^2^ area lit by blue light (λ470 nm). Once the larva had completely left the lit area, it was returned into the area. (G) Escape assay of *nsyb-QFDBD*, *nsyb-QFAD*, QUAS-ChR2 vae (red) and *nsyb-QFDBD*, *nsyb-QF2^w^AD*, QUAS-ChR2 larvae (blue), with or without *tub-QS* and quinic acid (QA). (H) Adult *nsyb-QFDBD*, *nsyb-QFAD*, *QUAS-shi^TS^* (red diamonds) and *nsyb-QFDBD*, *nsyb-QF2^w^AD*, *QUAS-shi^TS^* (dark-blue upward triangles) flies were paralyzed when placed in a 33° incubator at *t* = 0 min. Flies that also had a *tub-QS* transgene (yellow squares and light-blue downward triangles) were not paralyzed. The data show the average number of flies (out of 10, ± SEM) at the bottom of the vial over time. Each graph is an average of *n* = 5 repeats, apart from “QF2^w^AD+QS,” with *n* = 4. Red and blue dots indicate the time point when the corresponding genotypes with and without QS became significantly different for the first time (Student’s *t*-test with Holm–Sidak correction for multiple comparisons). Stars indicate data points where *nsyb-QFDBD*, *nsyb-QFAD*, *QUAS-shi^TS^* and *nsyb-QFDBD*, *nsyb-QF2^w^AD*, *QUAS-shi^TS^* flies performed significantly differently (Student’s *t*-test with Holm–Sidak correction for multiple comparisons).

Other *Drosophila* stocks used in this paper were acquired from the Bloomington *Drosophila* Stock Centre (indicated by # below) or were in personal stocks of the authors. Figure 1: QUAS-mCD8-GFP (#30003), tub-QS (#52112), nsyb-QF2 (attp2, personal stocks, O.R.), nsyb-QF2^w^ (#51960), and QUAS-Ppyr/Luc (#64773); Figure 2: UAS-mCD8-GFP (personal stocks, O.R.), elav-GAL4DBD (derived from #23868), VT019838-GAL4DBD (#75177), ChAT-GAL4DBD (#60318), UAS-Luc (#64774), 13xLexAop2-mCD8-GFP (#32204), VT007395-ZpLexADBD (personal stocks, B.J.D.), and VT009847-ZpLexADBD (personal stocks, B.J.D.); Figure 3: nsyb-LexAQF (#51953), 13xLexAop2-KZip+ (#76253), VGlut-GAL4DBD (#60313), tub>QS> (#77125), GH146-FLP (gift from Christopher Potter, Johns Hopkins University), 20C11-FLP (#55766), UAS-ChR2 (gift from Stefan Pulver, St Andrews), VGlut-GAL4 (#60312), 10xQUAS-ChR2 (#52260), and QUAS-shibire^TS^ (#30012); and Supplemental figures: R19F06-GAL4DBD (#69098), R53D01-GAL4DBD (#69075), VT059695-GAL4DBD (#73750), VT037031-ZpLexADBD (personal stocks, B.J.D.), and VT043690-ZpLexADBD (personal stocks, B.J.D.).

### Immunohistochemistry and confocal imaging

Dissection and immunostaining of adult brains was done as described previously (Riabinina *et al.* 2015). Briefly, on day 1, brains of 5–7 d.o. (day old) adult flies were dissected in ice-cold PBS, fixed at room temperature (RT) for 20 min in 4% paraformaldehyde in PBS + 0.3% Triton (PBT), then washed in PBT at RT for 1.5–6 hr, blocked in 5% normal goat serum (NGS) in PBT for 30 min, and placed in primary antibody mix at 4° for 3 nights on a shaker. On day 4, brains were washed in PBT at RT for 5–6 hr and placed in secondary antibody mix for 2 nights at 4° on a shaker. On day 6, brains were washed in PBT for 5–6 hr and left overnight in ∼50 μl of Vectashield mounting solution without shaking. On day 7, brains were mounted in Vectashield on a microscope slide. The primary antibody mix contained rabbit anti-GFP (#A11122, 1:100; Invitrogen, Carlsbad, CA), mouse nc82 (Developmental Studies Hybridoma Bank, 1:25), and 5% NGS in PBT. The secondary antibody mix contained Alexa Fluor 488 goat anti-rabbit (#A11034; Invitrogen), Cy3 anti-mouse (#115-165-062; Jackson Immunoresearch), and 5% NGS in PBT.

Images were acquired as z-stacks using a Leica (Wetzlar, Germany) SP8 upright confocal microscope equipped with an HCX IRAPO L25x/0.95W water-immersion objective (506323), at 512 × 512 pixel resolution with 1 μm z steps. LAS X v3.5.2 software was used for image acquisition. Imaging settings (laser intensity, gain, *etc*.) were kept identical for groups of images that were compared to one another. Images were processed by taking the maximum intensity projection, rotating, and recoloring in FIJI. Images shown are representative of three-to-five staining experiments for every genotype.

### Whole-animal imaging

Third-instar larvae were placed on a microscope slide and briefly put into a freezer to immobilize them. Images were taken on a Leica MZ10F zoom fluorescence microscope equipped with a Leica DFC 420C camera, QImaging LED light source, and LAS v.4 software. The white balance was adjusted automatically by taking an image of a white sheet of paper before experimental images. Identical settings were used to take images that were compared to each other. Images shown are representative of three to five experiments for every genotype.

### QA feeding

For larval experiments, gravid females were allowed to lay eggs in vials containing standard fly medium, supplemented with QA, and larvae remained in the vials until they reached the wall-climbing third-instar stage. For adult experiments, flies were raised on standard fly medium and were transferred into vials with QA at 2–3 d.o. for 5 days, at which point they were dissected. To make QA stock, 8 g of QA (#138622; Sigma [Sigma Chemical], St. Louis, MO) was dissolved in 40 ml ddH_2_O and adjusted to pH 7 with 5 M NaOH, bringing the total stock volume to 50 ml. Next, 1.6 ml/vial of this solution was thoroughly mixed into standard fly medium for larval or adult experiments.

### Luciferase assay

Each experiment assayed 9–30 larvae or 9–15 adult flies per genotype in groups of three. Third-instar larvae or 1–2 d.o. adult flies were placed in a 1.5 ml Eppendorf tube and stored at −80° until all samples for a given experiment were collected. A Dual-Luciferase Reporter Assay system (E1910; Promega, Madison, WI) was used for the experiments. Samples were homogenized in 200 μl of passive lysis buffer (E194A; Promega) per tube and kept on ice for ≥ 10 min. Then, the tubes were centrifuged for 5 min at 13,400 rpm and the supernatants transferred to new tubes. Next, 30 μl of supernatant from each tube were mixed with 30 μl of luciferase assay substrate (E151A; Promega), reconstituted in luciferase assay buffer (E195A; Promega) per well of a 96-well plate, and luminescence was measured immediately on a TECAN GENios plate reader, running XFluor 4 macros for Excel. We used 300-msec exposure for adult samples and 600 msec exposure for larval samples. We collected 3–10 measurements per experiment per genotype. The luciferase luminescence values were normalized by the amount of protein contained in the samples, to account for possible differences in the sizes of larvae and adults. For protein assay, 1.5 μl of supernatant was mixed with 100 μl of protein assay reagent (#500-0006; Bio-Rad, Hercules, CA) and light absorbance measured after 20 min on a FLUOstar Omega plate reader (BMG LABTECH) running Omega software v. 1.3. Two independent samples were measured per supernatant tube. The absorbance values were converted into milligrams per milliliter of protein by measuring a calibration curve with BSA dilutions (#B90015; New England Biolabs, Beverly, MA). All relative luminescence (RL) data points presented on the graphs (Figure 1, C and D, Figure 2, B and C, and Figure 3, A and B) were calculated as follows:

$$RL=\frac{LuciferaseMeasurement}{DerivedProtein},\;\;$$

where

$$DerivedProtein=30\left(a\left(\frac{ProteinMeasurement_{1}+ProteinMeasurement_{2}}{2}-BlankMeasurement\right)+b\right),$$

*a* and *b* parameters were obtained from the best linear fit to the calibration curve, plotted as [(average of three calibration measurement for a given dilution of BSA)-blank measurement] *vs.* [dilution of BSA in milligrams per milliliter]. Per genotype, four to six independent RL values were collected in each experiment. The genotypes are presented in the figures as mean ± SEM, and were compared with one- (larvae) or two- (adults) way ANOVA with Sidak’s multiple comparisons test.

We have observed significant differences between the measurements of adult males and females for some genotypes, arising from a consistently higher amount of protein per adult female. These differences were never observed for male and female larvae (data not shown). Thus, we present adult data separately for males and females.

### Larval whole-cell patch-clamp recordings

Larvae were grown in the dark on standard fly medium, supplemented with 100 µl/vial of 0.1 M all *trans*-retinal (#R2500; Sigma) in 100% EtOH. Recordings were performed at RT (20–22°). Third-instar larvae were dissected in external saline (135 mM NaCl, 5 mM KCl, 4 mM MgCl_2_·6H_2_O, 2 mM CaCl_2_·2H_2_O, 5 mM N-Tris(hydroxymethyl)methyl-2-aminoethanesulfonic acid, and 36 mM sucrose, pH 7.15). For each larva, the CNS was removed and secured to a Sylgard-coated (Dow-Corning, Midland, MI) cover slip using tissue glue (GLUture; WPI, Hitchin, UK). The glia surrounding the CNS were partially removed using protease (1% type XIV; Sigma) contained in a wide-bore (15 μm) patch pipette. Whole-cell recordings were carried out using borosilicate glass electrodes (GC100TF-10; Harvard Apparatus, Edenbridge, UK), fire-polished to resistances of between 10 and 14 MΩ. The aCC/RP2 motoneurons were identified by soma position within the ventral nerve cord. When needed, cell identity was confirmed after recording by filling with 0.1% Alexa Fluor 488 hydrazide sodium salt (Invitrogen), included in the internal patch saline (140 mM potassium gluconate, 2 mM MgCl_2_·6H_2_O, 2 mM EGTA, 5 mM KCl, and 20 mM HEPES, pH 7.4). Mecamylamine (1 mM, M9020; Sigma) was included in the external saline to block endogenous excitatory cholinergic-mediated currents to aCC/RP2 motoneurons and neuronal depolarization was elicited through UAS-ChR2 (Pulver *et al.* 2009) (λ470 nm, 500 msec, light intensity 9.65 mW/cm^2^ before reaching the LUMPlanFI 60x/0.9W Olympus objective) expressed in all motoneurons by the VGlut promoter. Recordings were made using a MultiClamp 700B amplifier. Cells were held at −55 mV, and recordings were sampled at 20 kHz and low-pass filtered at 10 kHz using pClamp 10.6 (Molecular Devices, Sunnyvale, CA). Only neurons with an input resistance of ≥ 500 MΩ were accepted for analysis. Eight recordings were taken per cell, average action potential number was calculated per 500 msec light pulse. Data in Figure 3E are presented as mean ± SEM, and were compared with a one-way ANOVA with Sidak’s multiple comparisons test.

### Larval escape assays

Individual third-instar larvae were assayed at RT (20–22°) in a 9-cm Petri dish that contained a thin layer of 1% agarose to prevent desiccation. The Petri dish was placed under a Leica MZ16F zoom fluorescence microscope with a Plan 1.0× lens, fluorescence light source, and a GFP filter cube (λ470 nm). Light intensity measured 9.87 mW/cm^2^ when completely zoomed out. Zoom 5 was used for experiments. Larvae were filmed using a uEye UI-233xSE-C camera with uEye Cockpit software, and data were stored in *.avi format. Each larva was allowed to crawl in the Petri dish for 2 min, before it was placed for 2 min in a 113-mm^2^ area illuminated by blue light. Wild-type larvae naturally avoid bright blue light and crawl away; however, larvae with ChR2 expressed in motoneurons (Figure 3F) or pan-neuronally (Figure 3G) are impaired in their ability to escape. A larva was returned into the blue-light area immediately after it had completely left the illuminated area. We counted the number of escapes during a 2-min period. Per genotype, 7–15 larvae were assayed. The data are shown as mean ± SEM. The genotypes were compared with one-way ANOVA with Sidak’s multiple comparisons test.

### Adult behavioral assay

Adult male and female 5–7 d.o. flies were assayed in groups of 10 (*N* = 4–5 groups per genotype) in clean empty standard fly vials. Flies were placed in a cooled incubator set to 33°, and video-recorded at 5 frames per second using a uEye camera UI-233xSE-C, controlled by uEye Cockpit software. The data were stored in *.avi format. The number of flies on the bottom of each vial was manually counted at 30-sec intervals. The data are shown as mean ± SEM, and were analyzed with multiple Student’s *t*-tests with Holm–Sidak correction.

### Data availability

Fly strains generated in this study are available from the Bloomington *Drosophila* Stock Centre and upon request from the corresponding author. Plasmids generated in this study are available upon request from the corresponding author. Supplemental material available at https://doi.org/10.25386/genetics.7801160.

## Results

### Quantification of strength of split-QF transactivators

To make the split-QF system compatible with existing split-GAL4 lines, we used the same leucine zippers (Pfeiffer *et al.* 2010). We attached Zip− to QFDBD and Zip+ to QFAD, defining the domains as previously reported (Riabinina *et al.* 2015), and expressed these transgenes under control of the neuronal synaptobrevin promoter *nsyb* (Figure 1A), integrating them in attp40 (DBD) and attp2 (AD) sites. We also generated QF2^w^AD::Zip+ flies (in attp2) that, similar to QF2^w^ transactivator (Riabinina *et al.* 2015) (Figure 1A), carry a mutated C-terminal with reduced negative charge and reduced activity. As expected, animals carrying *nsyb-QFDBD (attp40)*, *nsyb-QFAD (attp2)*, and *QUAS-mCD8-GFP* showed strong GFP expression throughout their nervous system (Figure 1B). This expression was repressible by *tub-QS* and inducible by QA (Supplemental Material, Figure S1). Similar, but weaker, expression was observed with *nsyb-QFDBD* and *nsyb-QF2^w^AD* (Figure 1B). Both split transactivators appeared to have lower activity than QF2 and QF2^w^ (Figure 1B).

To quantify the relative strength of split transactivators, and to compare QFAD and QF2^w^AD to existing p65AD and GAL4AD, we generated *nsyb-p65AD* (attp2) and *nsyb-GAL4AD* (attp2) flies, and expressed UAS-luciferase in larvae and adults (Figure 1, C and D, and Tables S1 and S2). We analyzed male and female flies separately due to the significant differences between sexes that were observed for some genotypes. No such differences were observed for larvae (data not shown). The strength of the QFDBD+QFAD transactivator was 2.2 times (*P* < 0.0001) lower than QF2 in larvae and 1.8–2 times (*P* < 0.0001) lower than QF2 in adults. Similarly, QFDBD+QF2^w^AD was three times (*P* < 0.0001) weaker than QF2^w^ in larvae and 1.4–2.6 times weaker in adults (*P* = 0.19 in females and *P* < 0.0001 in males). While relative expression levels varied between larvae (nonsexed) and male *vs.* female adults, QFAD was almost two times (*P* < 0.01) stronger than QF2^w^AD, and almost two times (*P* < 0.0001) weaker than p65AD. The GAL4AD was consistently weak. *tub-QS* provided strong repression of all original and split QF variants. We quantified the effect of QA derepression in larvae only, because in the adult brain QA is effective only in sensory receptor neurons and the pars intercerebralis neurons (Riabinina *et al.* 2015), presumably due to difficulty crossing the glial blood–brain barrier (Edwards and Meinertzhagen 2010). QA feeding to *tub-QS*, *nsyb-QFDBD*, *nsyb-QFAD (QF2^w^AD)* larvae, which otherwise had very low expression, resulted in restoration of expression to levels not significantly different from those of *nsyb-QFDBD*, *nsyb-QFAD (QF2^w^AD)* larvae (*P* = 0.87 and *P* = 0.62, respectively). In *tub-QS*, *nsyb-QF2 (QF2^w^)* larvae, the expression was restored to 50–60% of unrepressed levels (*P* < 0.0001 and *P* = 0.0031, respectively). These experiments demonstrate that the split-QF is fully functional, repressible, and inducible, due to the strong activity of the QFAD and QF2^w^AD activation domains.

### Quantification of split-QF transactivators when used with split-GAL4 and split-LexA

Next, we asked whether QFAD and QF2^w^AD may be effectively used together with existing GAL4DBD lines to provide a QS-repressible and QA-inducible alternative to the currently used p65AD. Pan-neuronal expression in the larval CNS, driven by *elav-GAL4DBD* and *nsyb-QF2/QF2^w^AD*, is strong, repressible, and inducible (Figure 2A, top). To investigate expression in the adult brain, we used *GAL4DBD* lines from the Janelia and Vienna collections, in combination with *nsyb-QFAD* and *nsyb-QF2^w^AD*, to drive GFP expression in antennal lobes, suboesophageal zone (SEZ), antennal mechanosensory and motor centers, optic lobes, and sparsely in other areas of the brain (Figure 2A, bottom and Figure S2). The observed expression was strong and repressible in all neurons in the predicted expression patterns, and QA-inducible in the olfactory and gustatory receptor neurons, as observed previously with *nsyb-QF2* (Riabinina *et al.* 2015). To quantify the strength of expression, we used *elav-GAL4DBD* in combination with the AD variants to drive luciferase in the CNS of third-instar larvae; however, the *elav-GAL4DBD*, *nsyb-p65AD* combination was lethal (Figure 2B and Table S3). Similarly to experiments in Figure 1C, QFAD-induced expression was not significantly different from QF2^w^AD (*P* = 0.16). In contrary to experiments with split-QF (Figure 1C), here, QA resulted in restoration of expression to ∼20–35% of that of the unrepressed split transactivators (*P* < 0.0001). Similarly to QF2 and QF2^w^, QA feeding restored expression levels of *tub-QS*, *nsyb-GAL4QF* to 60% of the unrepressed levels (*P* < 0.0001). To quantify expression levels in the adult CNS, we used *ChAT-GAL4DBD* to target cholinergic neurons and to avoid larval lethality, previously observed with *elav-GAL4DBD*, *nsyb-p65AD* (Figure 2C and Table S4). QFAD-driven expression was comparable with QF2^w^AD (*P* > 0.99) and almost four times weaker than p65AD (*P* < 0.0001). As previously observed (*e.g.*, Figure 1, C and D), *tub-QS* provided strong repression that did not differ from DBD- or AD-only controls (*P* > 0.99). These experiments demonstrate that QFAD and QF2^w^AD activation domains may be used together with GAL4DBD lines to provide a repressible and inducible, albeit weaker, alternative to p65AD.

The QFAD and QF2^w^AD activation domains also work with split-LexA reagents in the larval and adult CNS (Figure 2D and Figure S3). Moreover, expression is again both repressible and QA-inducible. Although we did not quantify the strength of expression by luciferase assay (due to the unavailability of a LexAop-Luc reporter), it appears that the QF2^w^AD domain works as well, or better, than QFAD in these experiments.

### Applications of split-QF

First, we asked how the QS repression compares with Killer-Zipper (Dolan *et al.* 2017), a tool that silences split-GAL4 expression by driving GAL4DBD-Zip+ construct with the LexA/LexAop system (Figure 3, A and B and Table S5). We observed that QS-induced repression was stronger than (*P* = 0.0071 for *nsyb-QFDBD*, *nsyb-QFAD*, *KZip* *vs.* *tub-QS*, *nsyb-QFDBD*, *nsyb-QFAD* females) or the same (all other genotypes, *P* > 0.83) as a Killer-Zipper-induced equivalent. The use of QS for repression is thus more advantageous than Killer-Zipper because it requires fewer transgenes and does not recruit the LexA/LexAop system. In addition, the mechanism of QS repression is different from that of Killer-Zipper, which is based on competitive dimerization between GAL4DBD-Zip+ and GAL4DBD-Zip− components.

Next, we tested whether the split-QF system may be effectively used for simultaneous expression of UAS and LexAop transgenes, and for advanced intersectional expression. We confirmed that a QF2^w^AD domain, when combined with GAL4DBD or ZpLexADBD, drives simultaneous expression from both *UAS-red fluorescent protein* and the *LexAop-GFP* reporters in both larvae and adults (Figure 3C). For advanced intersectional experiments, we regulated the expression of QS via the FLP-FRT system that, in turn, controlled the split transactivators. As expected, intersection of *Chat-GAL4DBD*, *nsyb-QF2^w^AD*, and *GH146-FLP* resulted in strong labeling of cholinergic olfactory projection neurons (Figure 3D, left). No labeling was observed when *Chat-GAL4DBD* was replaced by the glutamatergic driver *VGlut-GAL4DBD* (not shown). Similarly, we observed expression throughout the brain and in optic lobes in the cholinergic, but not glutamatergic (not shown), neurons that are targeted by *20C11-FLP* (Chen *et al.* 2014) (Figure 3D, middle). Interestingly, intersection of *VT009847-ZpLexADBD*, *nsyb-QFAD*, and *20C11-FLP* resulted in labeling of only one SEZ neuron (Figure 3D, right). These experiments demonstrate that split-QF can effectively achieve simultaneous and intersectional expression, narrowing down the expression patterns of split-GAL4, split-LexA, and FLP lines.

Finally, we applied the split-QF system to study physiology and behavior in *Drosophila*. To explore the usability of GAL4DBD + QFAD for electrophysiology, we performed whole-cell patch-clamp recordings from aCC and RP2 motoneurons of third-instar larvae. Neuronal depolarization was evoked through activation of UAS-ChR2 (Pulver *et al.* 2009) expressed in all motoneurons by *VGlut-GAL4DBD*, *nsyb-QF2^w^AD* or, in controls, *VGlut-GAL4* (Figure 3E and Table S6). The number of action potentials produced from *VGlut-GAL4DBD*, *nsyb-QF2^w^AD* larvae (42 ± 6 per 500 msec) was not different from that observed in the GAL4 controls (51 ± 6, *P* = 0.62). QS completely eliminated ChR2-induced depolarization in *tub-QS,VGlut-GAL4DBD*, *nsyb-QF2^w^AD* larvae (Figure 3E), while feeding larvae of the same genotype with QA partially restored depolarization and action potential count (10 ± 5), but to a level significantly below the unrepressed levels of *VGlut-GAL4DBD*, *nsyb-QF2^w^AD* larvae (*P* = 0.0016). These readouts of cellular activity are paralleled by behavioral phenotypes. We counted how many times (in 2 min) larvae of these four genotypes escaped a blue-light area (Figure 3F and Table S6). As expected, larvae containing the QS transgene escaped most readily (11 ± 1.8 escapes), while feeding larvae with QA significantly reduced the number of escapes to 9.3 ± 1.3 (*P* = 0.038), due to the seizure-like neuronal activity elicited by ChR2 activation. *VGlut-GAL4DBD*, *nsyb-QF2^w^AD* were also able to escape (5.9 ± 0.6), but significantly less than the QS larvae (*P* < 0.0001). *VGlut-GAL4* control larvae were unable to escape (0.2 ± 0.1).

We used the same assay to measure larval escape following activation of ChR2 driven pan-neuronally by split-QF (Figure 3G and Table S7). Abolished mobility was observed in larvae that expressed ChR2 (0 ± 0 escapes in *nsyb-QFDBD*, *nsyb-QFAD* and *nsyb-QFDBD*, *nsyb-QF2^w^AD* larvae*)*, and in larvae that expressed QS and were fed with QA (0.3 ± 0.2 and 0.1 ± 0.1 escapes for QFAD and QF2^w^AD, respectively). By contrast, QS-expressing larvae not fed with QA readily escaped the blue-light area (7.4 ± 0.7 and 8.0 ± 0.8 escapes, respectively).

We also assayed adult flies with pan-neuronal expression of *shibire^TS^* (Figure 3H and Table S8). When placed at 33°, *nsyb-QFDBD*, *nsyb-QFAD* flies became gradually paralyzed as expected. The same effect was observed in *nsyb-QFDBD*, *nsyb-QF2^w^AD* flies but took longer to develop, presumably due to the lower expression levels of *shibire^TS^*. When the expression of *shibire^TS^* was suppressed by *tub-QS*, no paralysis was observed.

Collectively, these experiments demonstrate that split-QF may be used with or without split-GAL4 to direct the expression of effectors in electrophysiological and behavioral assays.

## Discussion

Here, we demonstrate the use of split-QF by itself, or in combinations with split-GAL4 and split-LexA, for advanced intersectional experiments, concurrent independent use of UAS and LexAop transactivators, electrophysiology, optogenetics, and thermogenetics in *Drosophila*. Ultimately, split-QF facilitates targeting of small populations of cells, and may be used for neuronal connectomics analysis or to explore behavioral phenotypes that are produced by artificial activation of single neurons. The absence of large libraries of split-QF lines is currently limiting, but these lines may be generated from split-GAL4 and split-LexA stocks by, for example, the HACK method (Lin and Potter 2016).

### Quantification of transactivator activity

Three factors likely contribute to the lower strength of split transactivators when compared to the full-length ones (Figure 1, C and D and Figure 2, B and C). First, the *nsyb-QFDBD* transgene has been integrated into the attp40 site, which may be weaker than attp2, where the full transactivators are integrated. Second, the binding between QFDBD and QFAD (QF2^w^AD) is a process with a probability < 1, which is predicted to result in a lower number of reconstituted split transactivators compared to full-length ones. Consistent with this idea, the reporter expression by split transactivators appeared to be more variable than normally observed with full-length transactivators. Third, it is possible that the spatial configuration of the reconstituted QFDBD + QFAD (QF2^w^AD) is somewhat different and less efficient than the full-length QF2 (QF2^w^). Reduction of the transactivator strength is assessed by the expression level of reporters, which directly translates into the number of labeled cells as low levels of reporter expression could render some cells undetectable.

The luciferase and electrophysiology readouts of QA-fed GAL4DBD+QFAD/QF2^w^AD larvae were significantly below those of the nonsuppressed GAL4DBD+QFAD/QF2^w^AD larvae (Figure 2B and Figure 3E). On the other hand, GFP readouts of QA-fed LexADBD+QFAD/QF2^w^AD larvae and adults appeared to be equal to the unrepressed LexADBD+QFAD/QF2^w^AD (Figure 2D). These results, combined with the split-QF data (Figure 1C), indicate that the strength of QS binding to QFAD may be affected by the overall conformation of the reconstituted transactivator, subsequently resulting in different QA efficiency.

### Use of split-QF beyond *Drosophila*

The first use of split-QF was reported in *C. elegans* (Wei *et al.* 2012), albeit in a different form, including a QF dimerization domain, and without detailed characterization of QS and QA effects. Experiments presented here indicate that QF2^w^AD, QS, and QA are likely to be functional in *C. elegans*, and can extend the use of split-QF in this organism.

The Q-system works well across species. It has recently been introduced into zebrafish (Subedi *et al.* 2014) and malaria mosquitoes (Riabinina *et al.* 2016), dramatically expanding the very limited set of tools for transgene expression in mosquitoes. Split-QF may thus be a very useful addition to the genetic toolkit for these two organisms. Considering progressively wider use of genetic tools in other arthropods, such as moths (Long *et al.* 2015), beetles (Rylee *et al.* 2018), locusts (Wang *et al.* 2018), ants (Yan *et al.* 2017), and bees (Schulte *et al.* 2014), and in plants (Bruce *et al.* 2015), the split-QF and the Q-system may be the tools of choice in these organisms as well.

In summary, we present a split-QF system that is applicable for advanced anatomical, behavioral, and physiological manipulations in *Drosophila*. This system is fully compatible with and complementary to the existing split-GAL4 and split-LexA lines, and can greatly expand their use by making them QS-repressible and QA-inducible. In addition, combinations of split-QF with split-GAL4 and split-LexA systems can make extensive use of the available UAS and LexAop reporters.
